# Optic Neuropathy Following Acute Decompensated Hypothyroidism (Myxedema Crisis)

**DOI:** 10.7759/cureus.47793

**Published:** 2023-10-27

**Authors:** Dylan B McBee, Ping Hei A Lee, You Zhou, Saif Aldeen Alryalat, Andrew G Lee

**Affiliations:** 1 School of Medicine, Baylor College of Medicine, Houston, USA; 2 School of Medicine, Newcastle University, Newcastle upon Tyne, GBR; 3 Department of Ophthalmology and Visual Sciences, The University of Texas Medical Branch, Galveston, USA; 4 Department of Ophthalmology, Blanton Eye Institute, Houston Methodist Hospital, Houston, USA

**Keywords:** hypotension, anemia, myxedema crisis, nonarteritic ischemic optic neuropathy, shock-induced ischemic optic neuropathy

## Abstract

Myxedema crisis (MC) refers to an unusual state of regulatory dysfunction precipitated by uncontrolled hypothyroidism. The pathogenic consequences of MC are broad and may contribute to significant bodily decompensation across multiple organ systems. However, shock-induced ischemic optic neuropathy (SION) in this setting has not been reported previously. Here, we present the case of a 76-year-old female with presumed bilateral optic neuropathy following abdominal surgery. The patient experienced a prolonged and complicated postoperative course in which she remained off supplemental levothyroxine. Subsequently, her clinical status deteriorated until she met diagnostic criteria for acute decompensated hypothyroidism (MC). Upon awakening from a comatose state, she reported significant vision loss. A neuro-ophthalmologic evaluation later confirmed significant constriction of her visual fields, optic disc pallor, and global retinal nerve fiber layer loss consistent with nonarteritic ischemic optic neuropathy.

## Introduction

Acute decompensated hypothyroidism (myxedema crisis (MC)) is a potentially life-threatening but uncommon complication of untreated hypothyroidism. Although multi-system organ failure, hypotension, and shock can occur in MC, optic neuropathy is a previously unreported complication in the English language ophthalmic literature.

Shock-induced ischemic optic neuropathy (SION) refers to vision loss in the setting of severe systemic hypotension with subsequent hypoperfusion of the optic nerve. SION represents a subtype of nonarteritic ischemic optic neuropathy and has previously been described after sudden and dramatic blood loss. Postoperative vision loss resulting from general surgical, neurosurgical, spine, and cardiac procedures have all been attributed to SION previously [1]. Here, we describe a case of presumed bilateral optic neuropathy following MC possibly related to SION.

## Case presentation

A 76-year-old white female presented to the emergency department with a two-day history of progressive abdominal pain. An abdominal computed tomography (CT) scan demonstrated an internal hernia complicated by small bowel obstruction. After failing conservative medical management, the patient underwent an uncomplicated exploratory laparotomy with hernia reduction and minimal blood loss. Her past medical history was significant for autoimmune hypothyroidism (Hashimoto's thyroiditis) treated with levothyroxine. In the weeks prior to admission, the patient described only partial adherence to her medication regimen. Immediately postoperatively, the patient had normal mentation without visual complaints.

Her postoperative course following abdominal surgery was complicated by a prolonged ileus, nausea, vomiting, and an inability to tolerate her usual oral medications. The patient remained off her usual supplemental levothyroxine for a total of 10 days postoperatively with likely weeks of suboptimal dosing prior to admission. 

Despite restarting oral levothyroxine, the patient began to experience worsening drowsiness by postoperative day 12. Shortly thereafter, she was found to have acute renal failure that required continuous intravenous fluid replacement therapy and later hemodialysis. Serial complete blood counts at this time showed progressive anemia with hemoglobin levels of 6.8 (12-16 g/dL). The patient's status declined further as she developed a generalized tonic-clonic seizure, transient hypotension (blood pressure = 87/55), and acute hypoxemic respiratory failure (arterial oxygen saturation (SaO2) = 86%). She required tracheostomy tube placement and remained significantly obtunded in a state of myxedema coma. Cranial CT was performed and showed no acute intracranial hemorrhage. Magnetic resonance imaging (MRI) of the brain and orbits demonstrated T2-weighted and fluid-attenuated inversion recovery (FLAIR) hyperintensity at the anterior column of the right fornix but was otherwise unremarkable.

Thyroid function studies at this time revealed a low free T4 of 0.7 (0.9-1.7 ng/dL) and elevated thyroid-stimulating hormone (TSH) of 40 (0.27-4.20 uIU/mL) consistent with acute decompensated hypothyroidism. The patient had a myxedema score of 75 and was started on intravenous levothyroxine and hydrocortisone [2]. On postoperative day 27, the patient regained consciousness, was extubated, and complained of blurry vision in both eyes. The patient was subsequently discharged to outpatient rehabilitation.

**Figure 1:**
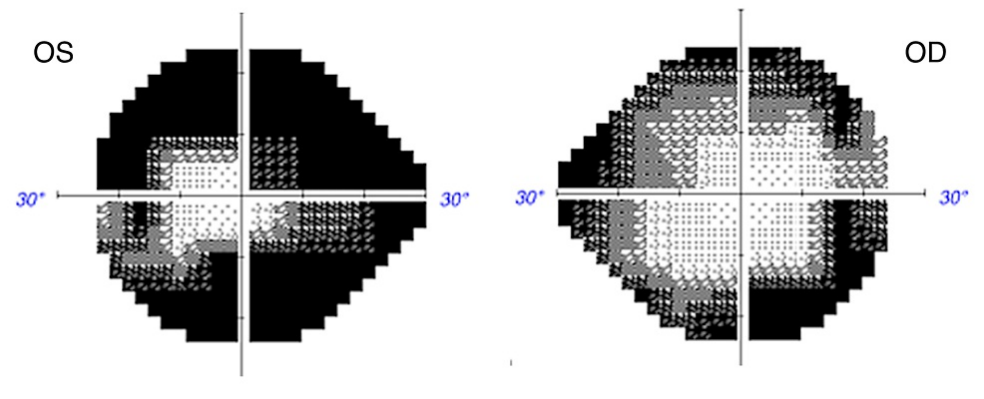
Automated perimetry (Humphrey visual field), performed three months after vision loss, demonstrates superior and inferior arcuate defects bilaterally OS: oculus sinister; OD: oculus dextrus

**Figure 2:**
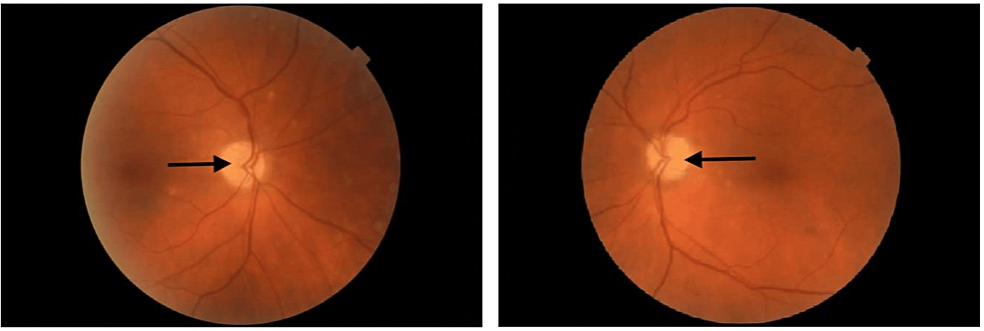
Fundus examination performed three months after vision loss with optic disc pallor bilaterally

Upon presenting to neuro-ophthalmology, her best-corrected visual acuity was 20/20 in the right eye (oculus dextrus (OD)) and 20/30 in the left eye (oculus sinister (OS)). Her pupils were isocoric with a left relative afferent pupillary defect. There were no motility deficits. Intraocular pressure measurements were 12 mm Hg OD and 13 mm Hg OS. Humphrey visual field testing demonstrated a mean deviation of -15.33 dB OD and -25.93 dB OS with dense superior and inferior arcuate defects and a constricted visual field oculus uterque (OU) (Figure 1). External and slit lamp examinations were unremarkable. Fundus examination showed optic disc pallor bilaterally with a cup-to-disc ratio of 0.1 OU (Figure 2). Optical coherence tomography demonstrated global retinal nerve fiber layer (RNFL) loss at 52 μm OD and 47 μm OS with temporal sparing. Table 1 shows the summary of patient laboratory values and clinical findings.

**Table 1:** Summary of patient laboratory values and clinical findings SaO2: arterial oxygen saturation; BP: blood pressure; TSH: thyroid-stimulating hormone; RNFL: retinal nerve fiber layer

Myxedema coma features
Precipitating factor (i.e., surgery)
Seizure
Transient hypoxemia (SaO2 = 86%)
Acute renal failure (hemodialysis required)
Decreased intestinal motility
Transient hypotension (BP = 87/55)
Laboratory values
Hemoglobin 6.8 (12-16 g/dL)
Free T4 0.7 (0.9-1.7 ng/dL)
TSH 40 (0.27-4.20 uIU/mL)
Pertinent ophthalmologic exam findings
Visual fields -15.33 dB OD -25.93 dB OS
RNFL 52 μm OD 47 μm OS
Cup-to-disc ratio 0.1 OD 0.1 OS
Optic disc pallor Present Present

Repeat MRI of the brain and orbits demonstrated resolution of the previously noted T2-weighted and FLAIR hyperintensity at the anterior column of the right fornix. The patient began low vision therapy and was seen for a six-month follow-up with modest improvement of her visual field defects.

## Discussion

SION is part of a larger spectrum of disease with varying etiologies, pathogeneses, and clinical features [1]. Admittedly, our patient's complicated postoperative course, prolonged altered mentation, and delayed fundoscopic examination make differentiating ischemic optic neuropathy subtypes challenging. However, several clues point to SION as the primary culprit of our patient's vision loss. Among other documented cases of SION, most visual deficits are bilateral, and nearly all occur in close succession with transient hypotension [1]. Compared with arteritic disease, preservation of a patient's visual acuity is more likely to occur [1]. Perimetry testing is often variable, yet relative rather than complete altitudinal defects are documented in other cases of SION [1]. Finally, delayed fundoscopic examination demonstrating optic disc pallor with a variable (often but not always >0.5) cup-to-disc ratio also supports our diagnosis.

MC is a form of decompensated hypothyroidism characterized by multi-system organ dysfunction [2]. A verified myxedema score may be used to distinguish the overlapping symptomatology of MC from other common medical conditions while predicting mortality in patients. The composite score accounts for precipitating factors, biochemical abnormalities, and clinical signs and symptoms seen in decompensated hypothyroidism [2]. After accounting for an identifiable precipitating factor (i.e., surgery), the presence of seizures, hypoxemia, diminished GFR, and decreased intestinal motility, our patient had a composite score of 75 [2]. This score is highly suggestive of MC with a predicted mortality rate of nearly 50% [2].

Cardiovascular dysfunction in the setting of MC is common [2]. Our patient experienced at least one reported episode of transient hypotension immediately following her seizure that likely resulted in hypoperfusion of the optic nerve. In addition, hemodialysis-related ischemic optic neuropathy has been documented previously and likely results from a combination of intradialytic hypotension, toxin accumulation, and anemia [3]. Furthermore, our patient experienced acute-on-chronic anemia in addition to acute hypoxemic respiratory failure. As such, inadequate oxygen delivery to the bilateral optic nerves likely occurred. Even metabolic derangement in the setting of renal failure is hypothesized to disrupt vascular autoregulation and thereby reduce compensatory blood flow to the optic nerves [4]. Finally, our patient's age as well as her history of chronic anemia and vasculopathic risk factors (i.e., hyperlipidemia and arterial hypertension) made her particularly susceptible to presumed SION [1].

## Conclusions

Ultimately, clinicians should be aware that acute decompensated hypothyroidism (MC) may precipitate vision loss and secondary optic neuropathy from multiple pathogenic mechanisms including MC-related hypotension, hypoxia, and metabolic derangement. MC can lead to coma (myxedema coma) and can be fatal. The prognosis depends on early recognition and treatment with parenteral levothyroxine.
